# Genome-Wide Identification, Characterization, and Expression Analysis of Small RNA Biogenesis Purveyors Reveal Their Role in Regulation of Biotic Stress Responses in Three Legume Crops

**DOI:** 10.3389/fpls.2017.00488

**Published:** 2017-04-25

**Authors:** Vanika Garg, Gaurav Agarwal, Lekha T. Pazhamala, Spurthi N. Nayak, Himabindu Kudapa, Aamir W. Khan, Dadakhalandar Doddamani, Mamta Sharma, P. B. Kavi Kishor, Rajeev K. Varshney

**Affiliations:** ^1^Center of Excellence in Genomics, International Crops Research Institute for the Semi-Arid TropicsHyderabad, India; ^2^Department of Genetics, Osmania UniversityHyderabad, India; ^3^School of Agriculture and Environment, The University of Western AustraliaCrawley, WA, Australia

**Keywords:** AGO, DCL, RDR, gene expression, biotic stress, Papillionidoids

## Abstract

Biotic stress in legume crops is one of the major threats to crop yield and productivity. Being sessile organisms, plants have evolved a myriad of mechanisms to combat different stresses imposed on them. One such mechanism, deciphered in the last decade, is small RNA (sRNA) mediated defense in plants. Small RNAs (sRNAs) have emerged as one of the major players in gene expression regulation in plants during developmental stages and under stress conditions. They are known to act both at transcriptional and post-transcriptional levels. Dicer-like (DCL), Argonaute (AGO), and RNA dependent RNA polymerase (RDR) constitute the major components of sRNA biogenesis machinery and are known to play a significant role in combating biotic and abiotic stresses. This study is, therefore, focused on identification and characterization of sRNA biogenesis proteins in three important legume crops, namely chickpea, pigeonpea, and groundnut. Phylogenetic analysis of these proteins between legume species classified them into distinct clades and suggests the evolutionary conservation of these genes across the members of Papillionidoids subfamily. Variable expression of sRNA biogenesis genes in response to the biotic stresses among the three legumes indicate the possible existence of specialized regulatory mechanisms in different legumes. This is the first ever study to understand the role of sRNA biogenesis genes in response to pathogen attacks in the studied legumes.

## Introduction

Among the legume crops, chickpea (*Cicer arietinum*), pigeonpea (*Cajanus cajan*), and groundnut (*Arachis hypogaea*) are the major crops catering to the needs of the underprivileged living in the semi-arid tropics of the world. Chickpea, being a rich source of carbohydrates, proteins, and vitamins, is of great importance from nutrition and fodder perspectives. With an annual global production of ~14.23 Mt, chickpea is ranked as the second most important legume after soybean (FAO, 2014). However, its production is vastly affected by several biotic stresses, including Ascochyta blight (AB) and Fusarium wilt (FW), which may cause grain yield and quality losses of up to 100% (Navas-Cortés et al., 2000; Pande et al., 2005). AB is regarded as the most destructive foliar disease of chickpea. It is known to have severe impact on crop yield and productivity in as many as 35 countries across six continents, with Australia being the most severely affected continent (Knights et al., 2003). The economic consequence of the disease is evident from the frequent epidemics occurring in many chickpea growing areas (Pande et al., 2005).

Pigeonpea is another important legume crop, which provides the protein in the low quality diets of people living in developing countries of Asia and Africa. Pigeonpea is the sixth most important legume food crop with global cultivation on ~5 M ha. In India, the crop is cultivated on 4.04 M ha with an annual production of about ~3 Mt (FAO, 2014). However, its yield has always been far below acceptable levels. Its stagnant yield plateau is mainly attributed to biotic stresses, such as FW and sterility mosaic disease (SMD; see Varshney et al., 2012). Infection caused by SMD in pigeonpea can cause losses up to 95–100% in plants <45 days old (Kannaiyan et al., 1984).

Groundnut, on the other hand, is mainly cultivated for its high quality edible oil and high protein content in the seed. It is an excellent cash crop, used in confectionery preparations, as well as cooking oil, and serves as a rich source of protein feed for animals. Cultivated groundnut is an allotetraploid (AABB), carrying half genome (AA) from *Arachis duranensis* and the other half (BB) from *Arachis ipaensis*. Groundnut is grown in more than 100 countries of Asia, Africa, and America with a global annual production of ~46 Mt (FAO, 2014). Groundnut productivity in India is a serious concern. Despite being one of the leading producers (~6.5 Mt), the productivity of groundnut in India is only 0.92 t/ha. Such low productivity has been attributed to the fungal foliar diseases, rust and late leaf spot (LLS), that generally occur together resulting in devastating yield losses (50–70%; Subrahmanyam et al., 1984; McDonald and Subrahmanyam, 1985).

Small RNAs (sRNAs) have emerged as one of the most versatile performers in the regulation of gene expression in both plants and animals, and also orchestrate the defense responses against several biotic stresses. sRNAs are involved in both transcriptional and post-transcriptional gene silencing. They are usually 20–24 nucleotides in length, and can be broadly classified into microRNAs (miRNAs) and short interfering RNAs (siRNAs) on the basis of their origin and biogenesis. sRNAs act at the core of RNA interference which has been implemented as a technology to not only understand the role of genes in plants but also to develop robust crops by gene manipulation. For example, genetic manipulation for virus resistance in plants has been achieved by silencing the coat protein (CP) gene utilizing the artificially synthesized exogenous sRNAs. Likewise, such approaches have also been used to improve the crop's nutritional quality and production (Kamthan et al., 2015). Therefore, the naturally synthesized endogenous sRNAs need more understanding, particularly in terms of their biogenesis and regulation of expression of target genes. The biogenesis and functioning of these sRNAs in plants are regulated by key proteins, such as Dicer-like (DCL), Argonaute (AGO), and RNA-dependent RNA polymerase (RDR). DCLs, also known as molecular rulers work as RNase III to cleave double stranded RNA molecules into short molecules of 21–24 nucleotides in length (MacRae et al., 2006). DCL proteins in Arabidopsis have been identified for their individual roles. DCL1 is responsible for miRNA generation, whereas DCL2, DCL3, and DCL4 are required for synthesis of siRNAs associated with virus defense, repeat associated siRNAs and trans-acting siRNAs, respectively (Liu et al., 2009). After being processed by the DCL proteins, the generated sRNAs are incorporated into RNA induced silencing complex (RISC). In RISC, the sRNA strands are separated and one of the strands is associated with AGO proteins, thus guiding it to the target for silencing (Meister and Tuschl, 2004). Besides DCL and AGO proteins, another set of proteins required for generation of sRNA are RNA Dependent RNA polymerases (RDRs). RDRs synthesize the double stranded siRNAs from single stranded RNAs by catalyzing the formation of phosphodiester bonds between ribonucleotides (Bartel, 2004). RDRs are also implicated in antiviral defense responses (Xie et al., 2001). DCL, AGO, and RDR proteins have been known to play an important role in the development and stress responses in a number of plant species, such as Arabidopsis (Xie et al., 2004), rice (Kapoor et al., 2008), maize (Qian et al., 2011), and foxtail millet (Yadav et al., 2015). These genes, if not directly implicated in combating stress, then at times act as a trigger leading to production of recently noted sRNA molecules which regulate the gene expression via epigenetic mechanisms.

Given the nutritional and economic importance of these legumes, and considering the constraints in yield and biomass imposed by biotic stresses, it is imperative to identify the potential sRNA biogenesis genes exploiting the recently available genome sequences of pigeonpea (Varshney et al., 2012), chickpea (Varshney et al., 2013), and groundnut (Bertioli et al., 2016; Chen et al., 2016). Moreover, chickpea, pigeonpea, and groundnut species belong to Galegoid, Phaseoloid, and Dalbergioid legume clades, respectively, the major clades of Papillionoideae sub-family. Therefore, genome-wide characterization and comparison of sRNA biogenesis genes could also provide insights into their evolution in legume crops.

## Materials and methods

### Identification and characterization of the DCL, AGO, and RDR genes

Two different approaches were used for genome-wide identification of the DCL, AGO, and RDR proteins: (i) In the first approach, the previously identified sRNA biogenesis protein sequences from Arabidopsis, rice, and soybean (Liu et al., 2014) were searched against the predicted gene models of chickpea, pigeonpea (http://ceg.icrisat.org/genomesequences.html) and groundnut (http://peanutbase.org/) using blastp program at an *E*-value threshold of 10^−5^; (ii) In parallel, the Hidden Markov Model (HMM) profiles of the domains with respect to DCL, AGO and RDR were downloaded from Pfam database and scanned against the predicted gene models of the legumes under study using Hmmer v 2.1.1 (Eddy, 2011).

A non-redundant set of putative sRNA biogenesis proteins identified from the two approaches were further confirmed for the presence of domains specific to each family using SMART and Pfam search. The identified genes were designated based on their phylogenetic relationship with their orthologs in Arabidopsis and soybean. The physio-chemical properties and subcellular localization of the identified proteins were calculated using ProtParam tool (web.expasy.org/protparam/) and ProtComp (www.softberry.com), respectively.

### Chromosomal distribution, gene structure, motif prediction, promoter, and miRNA analysis

The genomic coordinates of the above identified DCL, AGO, and RDR genes were used to map their locations on a physical map of the respective legume crops. The exon-intron structure of the genes were determined using the genome annotations which were further represented using Gene Structure Display Server (GSDS; Hu et al., 2015). The members were screened for conserved motifs using Multiple Em for Motif Elicitation (MEME) from MEME suite (Bailey et al., 2009) with a motif width of 10–200 and maximum number of motifs 20. The identified motifs were annotated using Pfam database (*E*-value cut-off 1.0). The 1,500 bp sequence upstream of the translation initiation codon of all DCLs, AGOs, and RDRs of all three legumes were scanned for the presence of *cis*-regulatory elements using PlantCARE database (Lescot et al., 2002). Only the *cis*-elements whose matrix score was more than five were considered. In parallel, the complete set of plant miRNAs reported in miRBase (Kozomara and Griffiths-Jones, 2014) were searched against the transcript sequences of DCL, AGO, and RDR of chickpea, pigeonpea, and groundnut using psRNATarget server (Dai and Zhao, 2011) with default parameters.

### Phylogenetic and gene duplication analysis, annotation, and interaction network studies

The deduced protein sequences of the identified DCL, AGO, and RDR genes of chickpea, pigeonpea, and groundnut along with their counterparts from Arabidopsis and soybean were subjected to phylogenetic analysis. Initially, the multiple sequence alignments of the protein sequences from each family were carried out using ClustalW. The alignments were manually corrected and then used to calculate a *p* distance matrix after pairwise deletions of gaps. Finally, the phylogenetic tree was constructed based on *p* distance matrix using Neighbor-Joining method implemented in MEGA6 (Tamura et al., 2013) with 5,000 bootstrap replicates.

The duplicated genes (paralogs) in each legume were identified using blastp search with an *E*-value threshold of 10^−5^ and 80% sequence identity. For the identification of orthologs, the DCL, AGO, and RDR proteins of chickpea, pigeonpea, and groundnut were searched against the complete protein set of Medicago and soybean using blastp program with an *E*-value threshold of 10^−10^ and sequence identity of more than 80%. The orthologous relationships were further represented using circos (Krzywinski et al., 2009). The DCL, AGO, and RDR protein sequences of chickpea, pigeonpea, and groundnut were annotated using Blast2GO (Conesa et al., 2005). Further, the protein-protein interaction studies were carried out using web-based STRING database (Szklarczyk et al., 2011) which assesses and integrates both physical and functional protein-protein associations. The protein sequences of DCL, AGO, and RDR of chickpea, pigeonpea, and groundnut were searched against Arabidopsis proteins and corresponding best hits were used for network association studies. For association studies, STRING extracts curated data from various databases such as GO, KEGG, and tries to establish possible interactions among the proteins.

### Plant materials and stress treatment

In chickpea, four AB responsive genotypes, two moderately resistant (ILC 3279 and ICCV 05530) and two susceptible (C 214 and Pb 7) were used for expression analysis at 7th and 11th day post-inoculation (dpi). Seedling raising and inoculum preparation were done as described earlier (Pande et al., 2011). Ten-day-old seedlings of both resistant and susceptible genotypes, acclimatized for 24 h to 20 ± 1°C temperature and 12 h of photoperiod in control environment facility, were sprayed with the *A. rabiei* spore suspension of 5 × 10^4^ conidia/ml until runoff. The leaf tissues were harvested on 7th (initial stress) and 11th (severe stress) dpi.

In the case of pigeonpea, three genotypes including one resistant (ICPL 20096) and two susceptible (ICPL 332-susceptible, ICPL 8863-highly susceptible) to SMD, were grown under glasshouse conditions (25 ± 2°C temperature, 16 h photoperiod). Seedlings emerging after 10 days of sowing were stapled with SMD-infected pigeonpea leaves with at least five live mites (Nene and Reddy, 1976). The first set of leaf samples was collected at 7th dpi as initial stage of stress, followed by the second sampling at 14th dpi as severe stress.

In the case of groundnut, two resistant (GPBD 4, ICGV 13208) and one susceptible (TAG 24) genotypes for rust and LLS were used in the study. ICGV 13208 was an introgression line with resistance contributed by genomic region from GPBD 4 (Varshney et al., 2014). The three genotypes were infected with both rust (*Puccinia arachidis*) and LLS (*Phaeoisariopsis personata*) pathogens. The 35-days-old plant leaves were uniformly sprayed with rust and LLS pathogens with a spore suspension of 5 × 10^4^ spores/ml using an atomizer under maintained high humid conditions for 24 h at 25°C. The leaf samples were collected at 21st, 35th, and 50th dpi. A control set was maintained without any inoculations for all the three crops. The harvested leaf samples were stored at −80°C until RNA isolation.

### Quantitative real time PCR (qRT-PCR) analysis

Total RNA from chickpea and groundnut leaf tissues was isolated using “NucleoSpin® RNA Plant” kit (Macherey-Nagel, Germany); and for pigeonpea, Plant RNA Miniprep kit, XcelGen (XG661-01, Xcelris, India) was used according to the manufacturer's instructions. The qualitative and quantitative assessment of these total RNA samples were conducted using Agilent 2100 Bioanalyzer (Agilent Technologies, CA, USA) and Nano Drop 8000 Spectrophotometer (Thermo Scientific, USA). The RNA samples with RNA integrity value of more than seven and 260/280 ratio of 1.8–2.1 were used to synthesize cDNA samples from the three biological replicates. The expression of sRNA biogenesis (DCL, AGO, and RDR) genes were studied using quantitative real time PCR (qRT-PCR). cDNA was prepared using SuperScript® III First Strand Synthesis System for RT-PCR (Invitrogen, CA, USA) according to the manufacturer's instructions. The gene specific primers were designed using PrimerQuest (Integrated DNA Technologies, http://www.idtdna.com) with default parameters for qRT-PCR (Supplementary Table 1). The qRT-PCR reactions were performed using SYBR green master-mix in 96 well-plates with three biological replicates and two technical replicates using Actin in pigeonpea, GAPDH in chickpea, and ADH3 in groundnut as the endogenous control. The PCR conditions used were as follows: 2 min at 50°C, 10 min at 95°C, and 40 cycles of 15 s at 95°C, and 1 min at 60°C. The relative transcriptional level in terms of fold-change was calculated using the 2^−ΔΔCT^ method and student's *t*-test was used to calculate significance (Livak and Schmittgen, 2001).

## Results and discussion

### Genome-wide characterization and phylogenetic study of DCL, AGO, and RDR proteins

**Figure 1:**
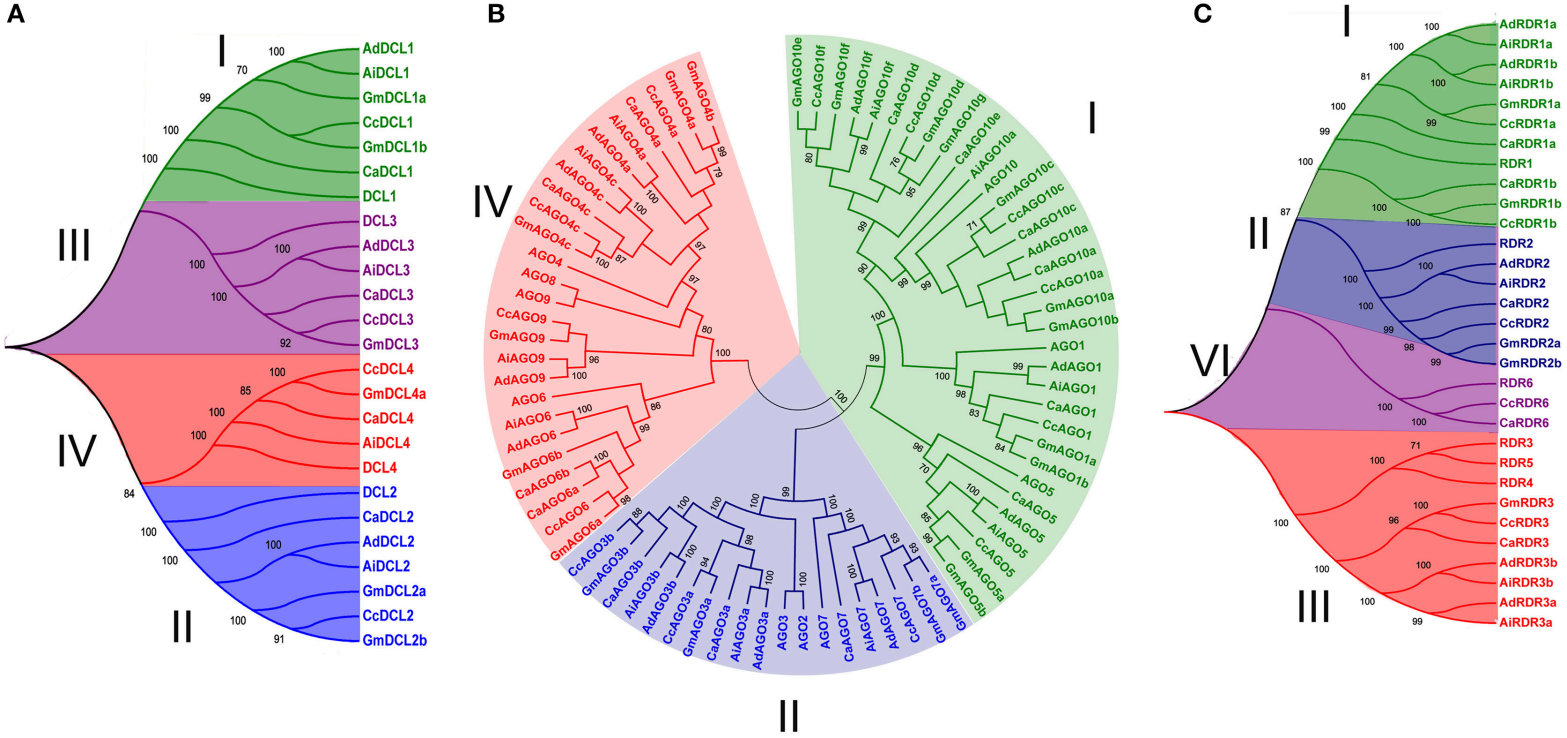
**Phylogenetic analysis of DCL, AGO, and RDR proteins in chickpea, pigeonpea, and groundnut**. The tree was constructed by Neighbor-joining method using orthologs from Arabidopsis and soybean for **(A)** DCL with four; **(B)** AGO with three; and **(C)** RDR with four clades. Bootstrap values ≥70 are indicated in the figure. Ca, Cc, Ad, and Ai represent chickpea, pigeonpea, *A. duranensis*, and *A. ipaensis*, respectively.

Using similarity and HMM searches, a total of four DCLs (DCL1, DCL2, DCL3, and DCL4) were found both in chickpea and pigeonpea similar to Arabidopsis (Xie et al., 2004). In the case of groundnut, three and four DCLs were found in *A. duranensis* and *A. ipaensis* respectively (Table 1). A higher number of DCLs in *A. ipaensis* can be attributed to a higher number of duplicated events in *A. ipaensis* in comparison to *A. duranensis* (Bertioli et al., 2016). Varied number of DCLs were reported in other crops *viz.*, rice (8), maize (5), soybean (7), sorghum (3), and foxtail millet (8) (Kapoor et al., 2008; Qian et al., 2011; Liu et al., 2014; Yadav et al., 2015). The phylogenetic analysis grouped DCLs into four clades depicted as DCL I, DCL II, DCL III, and DCL IV (Figure 1A). The DCLs ranged in length from 1,410 to 1,864 amino acid (aa) in chickpea, 1,203 to 1,913 aa in pigeonpea, 1,376 to 1,949 aa in *A. duranensis*, and 1,398 to 1,947 aa in *A. ipaensis*. Interestingly, in all three legumes the shortest length was shared by clade II DCLs. All DCLs were found to be localized in nucleus supporting the findings that sRNA precursors undergo nuclear processing in plants (Papp et al., 2003).

**Table 1:** **Physio-chemical properties and localization of DCL genes/proteins in chickpea, pigeonpea, and groundnut (*A. duranensis* and *A. ipaensis*)**.

**Gene**	**Locus name**	**Genome coordinates**	**Length (aa^#^)**	**Localization**	MW^*^	**Introns**
**CHICKPEA**						
CaDCL1	*Ca_01367*	Ca3:39793013:39804554 (−)	1,864	Nuclear	209706.6	20
CaDCL2	*Ca_08007*	Ca1:7525781:7537015 (−)	1,410	Nuclear	159948.3	23
CaDCL3	*Ca_01677*	Ca5:35534919:35547109 (−)	1,649	Nuclear	184754.8	24
CaDCL4	*Ca_06761*	Ca7:5953535:5974786 (−)	1,780	Nuclear	199784.1	28
**PIGEONPEA**						
CcDCL1	*C.cajan_10975*	CcLG03:29072965:29085159 (−)	1,913	Nuclear	215155.4	22
CcDCL2	*C.cajan_25625*	Scaffold000032:233152:246602 (−)	1,203	Nuclear	136996.2	17
CcDCL3	*C.cajan_29114*	Scaffold126331:336037:350387 (+)	1,553	Nuclear	173766.2	24
CcDCL4	*C.cajan_29904*	Scaffold135129:302372:316356 (+)	1,481	Nuclear	166998	23
***A. duranensis***						
AdDCL1	*Aradu.2PR08*	Aradu.A06:1660935:1671391 (+)	1,949	Nuclear	219036.7	21
AdDCL2	*Aradu.M1A8C*	Aradu.A04:9242477:9261536 (−)	1,376	Nuclear	157029.1	26
AdDCL3	*Aradu.EH11Z*	Aradu.A10:89258296:89268902 (−)	1,680	Nuclear	188027.6	25
***A. ipaensis***						
AiDCL1	*Araip.VEU15*	Araip.B06:20391007:20401632 (−)	1,947	Nuclear	218954.3	19
AiDCL2	*Araip.5RQ6Z*	Araip.B04:10697651:10706715 (−)	1,398	Nuclear	159701.5	23
AiDCL3	*Araip.0XV9H*	Araip.B10:112301476:112318201 (−)	1,692	Nuclear	189305.7	33
AiDCL4	*Araip.TBT76*	Araip.B01:134634628:134652430 (+)	1,642	Nuclear	185300.1	29

* *MW, Molecular weight in Daltons*.

# *aa, amino acid*.

A total of 13 AGO genes each in chickpea, pigeonpea and 22 (11 each from *A. duranensis* and *A. ipaensis*) in the case of groundnut were identified (Table 2). However, in an earlier study in Arabidopsis, a total of 10 AGOs were identified with AGO1 involved in miRNA and ta-siRNA biogenesis; AGO4 and AGO6 responsible for DNA methylation (Baumberger and Baulcombe, 2005). The length of identified AGOs varied from 776 to 1,094 aa in chickpea, 821 to 1,054 aa in pigeonpea, 854 to 1,033 aa in *A. duranensis*, and 792 to 1,056 aa in *A. ipaensis* (Table 2). The phylogenetic tree classified the AGO proteins into three clades: AGO I, AGO II and AGO IV in all the three legumes, in accordance with the findings in Arabidopsis (Xie et al., 2004), and soybean (Liu et al., 2014). Chickpea and pigeonpea shared six, three, and four members each in clades, AGO I, AGO II, and AGO IV, respectively, in contrast to groundnut where *A. duranensis* and *A. ipaensis* had four, three, and four members each in AGO I, AGO II, and AGO IV clades, respectively (Figure 1B). Phylogenetic analysis clustered AGO1, AGO5, AGO10 in clade I, AGO3, AGO7 in clade II, and AGO4, AGO6, AGO9 in clade IV in all three legumes. We did not find AGO2 and AGO8 homologs in any of the three legumes considered in this study. Further, all the AGO3 genes were found to cluster with AGO2 and AGO3 genes of Arabidopsis. Interestingly, we found the presence of two homologs of AGO3 (AGO3a and AGO3b) in all three legumes, similar to soybean (Liu et al., 2014) but in contrast to Arabidopsis (Xie et al., 2004). It can be postulated that the two homologs of AGO3 compensates for the absence of AGO2 in legumes. Absence of AGO8 protein in the legumes is in agreement with the findings in Medicago and soybean that also lacked AGO8 homologs (Capitao et al., 2011; Liu et al., 2014). This could be attributed to the loss of AGO8 during the course of speciation. Interestingly, chickpea clade IV AGO was marked by the presence of two AGO6 homologs (AGO6a and AGO6b). This second AGO6 homolog was absent in pigeonpea and peanut. Instead, these species possess one AGO9 homolog that was absent in chickpea implying the legume specific diversification of sRNA biogenesis genes. All members of clade I AGOs of chickpea, pigeonpea, and groundnut were predicted to be localized in cytoplasm supporting the findings that AGOs are known to be present in high concentrations in cytoplasm and interaction of AGO1 with miRNA occurs in the cytoplasm to facilitate the RISC to find its target mRNA. However, most members of clade II and IV were predicted to be localized in the nucleus.

**Table 2:** **Physio-chemical properties and localization of AGO genes/proteins in chickpea, pigeonpea, and groundnut (*A. duranensis* and *A. ipaensis*)**.

**Gene**	**Locus name**	**Genome coordinates**	**Length (aa^#^)**	**Localization**	MW^*^	**Introns**
**CHICKPEA**						
CaAGO1	*Ca_18035*	Scaffold1006:491140:497352 (+)	1,094	Cytoplasmic	120964.3	20
CaAGO3b	*Ca_07965*	Ca1:7035349:7040048 (−)	1,033	Extracellular	117036.9	2
CaAGO10d	*Ca_19437*	Ca1:33057594:33065348 (+)	867	Cytoplasmic	98730.4	20
CaAGO4a	*Ca_10233*	Ca2:32900469:32906134 (+)	906	Nuclear	101240.7	20
CaAGO7	*Ca_11683*	Ca2:13131307:13136219 (+)	1,016	Nuclear	116210.4	2
CaAGO10a	*Ca_04545*	Ca4:13222797:13228230 (+)	976	Cytoplasmic	110048.8	20
CaAGO10c	*Ca_15463*	Ca4:32128211:32135531 (−)	949	Cytoplasmic	106625.5	20
CaAGO4c	*Ca_07531*	Ca5:40020432:40030536 (+)	938	Nuclear	105263.4	22
CaAGO6a	*Ca_19212*	Ca5:41701444:41707829 (+)	776	Nuclear	86435	19
CaAGO6b	*Ca_19215*	Ca5:41677400:41685345 (+)	875	Nuclear	97419.7	22
CaAGO3a	*Ca_06544*	Ca6:19393544:19397617 (−)	887	Extracellular	100743.4	2
CaAGO5	*Ca_17448*	Ca6:29304385:29309715 (−)	966	Cytoplasmic	108127.8	21
CaAGO10e	*Ca_06629*	Ca7:7288639:7294813 (−)	894	Cytoplasmic	101081.6	21
**PIGEONPEA**						
CcAGO4a	*C.cajan_05692*	CcLG02:12753469:12759534 (−)	910	Nuclear	101927.4	21
CcAGO6	*C.cajan_05742*	CcLG02:13199198:13214696 (+)	897	Nuclear	100807.9	21
CcAGO10c	*C.cajan_06023*	CcLG02:15919454:15927414 (+)	934	cytoplasmic	105466.2	21
CcAGO10a	*C.cajan_07464*	CcLG02:31329417:31336496 (+)	953	Cytoplasmic	107637.4	21
CcAGO10d	*C.cajan_13892*	CcLG10:5900494:5908489 (+)	905	cytoplasmic	102982.4	21
CcAGO9	*C.cajan_14693*	CcLG10:15320919:15328269 (+)	885	nuclear	100148.7	21
CcAGO1	*C.cajan_15733*	CcLG08:3847540:3853508 (−)	1,054	cytoplasmic	116584.9	21
CcAGO7	*C.cajan_17999*	CcLG07:7367814:7372842 (−)	1,028	nuclear	117568.5	2
CcAGO5	*C.cajan_21615*	CcLG04:7177034:7182639 (+)	947	Cytoplasmic	106187.2	21
CcAGO4c	*C.cajan_23341*	CcLG05:2617913:2625461 (+)	945	Nuclear	106295.4	20
CcAGO10f	*C.cajan_26220*	Scaffold000017:700964:707596 (−)	905	Cytoplasmic	102649.3	21
CcAGO3b	*C.cajan_37379*	Scaffold134686:75450:78912 (−)	821	extracellular	93401.5	2
CcAGO3a	*C.cajan_42216*	Scaffold137616:114442:117800 (−)	845	Cytoplasmic	95803.9	1
***A. duranensis***						
AdAGO4c	*Aradu.67AL7*	Aradu.A04:621757:629400 (−)	925	Nuclear	103216.9	23
AdAGO4a	*Aradu.2V8U8*	Aradu.A07:5793658:5804159 (−)	913	Nuclear	102050.4	26
AdAGO10f	*Aradu.RY0TT*	Aradu.A01:102555563:102567085 (−)	886	Cytoplasm	100146	23
AdAGO3a	*Aradu.6K97H*	Aradu.A04:31982490:31985928 (+)	854	Cytoplasm	95879.1	2
AdAGO7	*Aradu.RF5XH*	Aradu.A10:8971874:8975054 (+)	910	Cytoplasm	103830.2	1
AdAGO9	*Aradu.FQ8VL*	Aradu.A10:107171742:107178558 (+)	936	Nuclear	105395	21
AdAGO6	*Aradu.E98LA*	Aradu.A10:108178610:108187445 (−)	915	Nuclear	102363.5	24
AdAGO1	*Aradu.R24S8*	Aradu.A05:98685962:98692253 (+)	1,033	Cytoplasm	114347	20
AdAGO5	*Aradu.W5GQC*	Aradu.A03:621532:627450 (−)	962	Cytoplasm	107195.8	21
AdAGO10a	*Aradu.X0U23*	Aradu.A09:110871552:110876942 (+)	929	Cytoplasm	104210.8	19
AdAGO3b	*Aradu.0YT2V*	Aradu.A04:7602305:7609000 (−)	1,027	Extracellular	116651.2	5
***A. ipaensis***						
AiAGO4c	*Araip.DM5GK*	Araip.B04:1151800:1159887 (−)	952	Nuclear	106574.8	23
AiAGO4a	*Araip.AEF9E*	Araip.B07:5421317:5431673 (−)	943	Nuclear	105474.7	26
AiAGO10a	*Araip.I8ABE*	Araip.B09:146081716:146087148 (−)	965	Cytoplasm	108274.8	19
AiAGO9	*Araip.A6QK5*	Araip.B10:133942144:133948674 (+)	880	Nuclear	99179.8	21
AiAGO1	*Araip.FPV8R*	Araip.B05:126022537:126029025 (−)	1,056	Cytoplasm	117204.3	21
AiAGO3b	*Araip.YRA5M*	Araip.B04:9235071:9241757 (−)	1,027	Extracellular	116573.1	5
AiAGO3a	*Araip.M1C18*	Araip.B04:30757796:30761710 (+)	951	Extracellular	106169.7	3
AiAGO7	*Araip.D30ND*	Araip.B10:14593307:14596492 (+)	910	Cytoplasm	103801.2	1
AiAGO10f	*Araip.Z6W66*	Araip.B10:7828151:7838826 (+)	792	Cytoplasm	89506	22
AiAGO6	*Araip.70L1G*	Araip.B10:134978071:134986384 (−)	918	Nuclear	102670.7	23
AiAGO5	*Araip.DFU9Y*	Araip.B03:2620052:2626164 (−)	966	Cytoplasm	107602.3	21

* *MW, Molecular weight in Daltons*.

# *aa, amino acid*.

Chickpea, pigeonpea, *A. duranensis*, and *A. ipaensis* encodes for five RDRs each. A similar number of RDRs (6) were identified in Arabidopsis responsible for the formation of double stranded RNA (dsRNA) molecules (Wassenegger and Krczal, 2006). Length of RDRs ranged from 987 to 1,228 aa in chickpea, 957 to 1,135 aa in pigeonpea, 795 to 1,131 aa in *A. duranensis*, and 839 to 1,116 aa in *A. ipaensis* (Table 3). Similar to previous reports (Xie et al., 2004; Kapoor et al., 2008; Qian et al., 2011; Liu et al., 2014), the phylogenetic analysis grouped chickpea and pigeonpea RDRs into four clades—RDR I, RDR II, RDR III, and RDR VI. Each clade contains one RDR gene per species except clade I that contained two homologs per species. By contrast, RDRs from groundnut were clustered into three clades each one containing two members except clade RDR II (Figure 1C). The presence of two homologs of RDR III (RDR 3a and RDR 3b) and absence of RDR VI in groundnut could be explained by incorporation or loss of these genes in early diverging Dalbergioid species which diverged from other Papillionidoids some 55–56 million years ago (Rispail and Rubiales, 2016).

**Table 3:** **Physio-chemical properties and localization of RDR genes/proteins in chickpea, pigeonpea, and groundnut (*A. duranensis* and *A. ipaensis*)**.

**Gene**	**Locus name**	**Genome coordinates**	**Localization**	**Length (aa^#^)**	MW^*^	**Introns**
**CHICKPEA**						
CaRDR1a	*Ca_15154*	Ca4:37163561:37169980 (–)	Extracellular	1,126	129266.8	3
CaRDR1b	*Ca_15153*	Ca4:37175471:37186589 (–)	Extracellular	1,228	140705.2	6
CaRDR2	*Ca_17314*	Ca7:9440293:9444640 (+)	Extracellular	1,122	127939.2	3
CaRDR3	*Ca_17876*	Ca8:15687606:15699452 (+)	Extracellular	987	111791.6	16
CaRDR6	*Ca_01765*	Ca5:34764715:34768405 (+)	Extracellular	1,070	122471.4	1
**PIGEONPEA**						
CcRDR1a	*C.cajan_29335*	Scaffold132418:90943:97761 (+)	Extracellular	1,126	128947.9	3
CcRDR1b	*C.cajan_29332*	Scaffold132418:64081:70327 (+)	Extracellular	1,135	130368.1	3
CcRDR2	*C.cajan_02456*	CcLG11:26891564:26896223 (+)	Extracellular	1,120	128072.7	3
CcRDR3	*C.cajan_41518*	Scaffold136224:56966:71567 (+)	Extracellular	957	108790.3	17
CcRDR6	*C.cajan_33988*	Scaffold135491:42338:46321 (+)	Extracellular	1,050	119800.6	2
***A. duranensis***						
AdRDR1a	*Aradu.FUK4V*	Aradu.A08:44261916:44269684 (+)	Extracellular	1,107	126425.2	4
AdRDR1b	*Aradu.V2M80*	Aradu.A09:83785988:83794273 (–)	Extracellular	1,112	127088	5
AdRDR2	*Aradu.0K8SM*	Aradu.A01:70282483:70286955 (–)	Extracellular	1,131	129260.7	3
AdRDR3a	*Aradu.A7LBJ*	Aradu.A05:89873064:89881688 (–)	Extracellular	795	90116	17
AdRDR3b	*Aradu.4GR15*	Aradu.A10:4871397:4880329 (+)	Extracellular	999	113701.6	17
***A. ipaensis***						
AiRDR1a	*Araip.2Y7ZS*	Araip.B08:123098782:123108524 (+)	Extracellular	1,107	126355.1	4
AiRDR1b	*Araip.QU4QG*	Araip.B09:102305651:102313983 (+)	Extracellular	1,116	127617.7	5
AiRDR2	*Araip.63JS7*	Araip.B01:100821579:100825755 (–)	Extracellular	839	95203.1	2
AiRDR3a	*Araip.TQ462*	Araip.B05:138855272:138866105 (+)	Extracellular	958	108703.5	18
AiRDR3b	*Araip.9W5SC*	Araip.B10:6934819:6943920 (+)	Extracellular	981	112385.6	18

* *MW, Molecular weight in Daltons*.

# *aa, amino acid*.

### Structural organization and conservation patterns

DCLs are large proteins with multiple domains, namely, DEAD, Helicase-C, Dicer-dimer, PAZ, two tandem Ribonuclease III, and double stranded RNA binding domain (dsrm). The characteristic DCL domain organization was found in all chickpea, pigeonpea, and peanut DCL homologs except CcDCL2, which lacks the DEAD domain. In the case of chickpea and groundnut, all DCLs contained two copies of dsrm domain except clade II DCLs. The clade II DCLs harbored a single copy of dsrm domain in accordance with clade II DCL proteins of Arabidopsis and soybean. Interestingly, in pigeonpea only clade I DCL was found to contain two copies of dsrm domain (Supplementary Figures 1A–C). The differences between sequences are shared by all species and correspond to DCL clade specific changes which might have a functional implication. Motif 1 (CY[QE]RLE[FY][LV]GD[AS]VLD) corresponding to RNase III domain was found among all the members of studied legumes. Another motif PK[AV]LGD[IL][VI]ES was seen in all members of chickpea, pigeonpea, and groundnut except for clade IV of groundnut. In the case of groundnut, motif 8 (WLPHDAPPSA), and motif 9 (EMQDFANEPENEEQPS) were seen to be specific for clade I and II, respectively, but were missing in both chickpea and pigeonpea (Supplementary Figures 1A–C). Exon-intron structure of DCL genes identified the maximum number of introns in clade IV in chickpea, clade III in pigeonpea, clade II in *A. duranensis*, and clade III in *A. ipaensis*. The number of introns ranged from 19 to 22, 17 to 26, 24 to 33, and 23 to 29 in DCL clade I, II, III, and IV, respectively (Supplementary Figures 1A–C).

AGOs are marked by the presence of four domains: N-terminal domain, PAZ, MID, and PIWI domain. MID domain is known to have a nucleotide specificity loop which makes it a recognition and binding center for sRNAs (Frank et al., 2012). In the current study, AGOs identified in the three legumes contained ArgoL1 (Argonaute linker 1 domain), ArgoL2 (Argonaute linker 2 domain), and Argomid (mid domain of Argonaute) domain beside the PIWI, PAZ, ArgoN (N-terminal domain of Argonaute) domain. An additional, Gly-rich_AGO1 domain was also found as a signature sequence of CcAGO1, CaAGO1, AdAGO1, and AiAGO1 proteins (Supplementary Figures 2A–C) in agreement with observations in Arabidopsis and soybean (Xie et al., 2004; Liu et al., 2014). In AGO family, motif 1 (PGTVVD[TS]KI[CT]HP) was found in all members of chickpea, pigeonpea, and groundnut. A conserved stretch of T[I/L]IFGADVTHP was seen in members of AGO I clade of all three species. This stretch was substituted by T[L/M]ILGMDVSHG in clade II and V[I/M] F[I/M]GADVTHP in clade III for both chickpea and pigeonpea. Motif 7, 16, and 20 in chickpea, motif 11, 12, and 13 in pigeonpea, and motif 6, 16, 18, and 19 in groundnut were shared by clade IV members only. Motifs 18 of chickpea, 17 of pigeonpea, and 8, 15 of groundnut were shared by members of clade II. Intronic organization identified 2–22, 1–21, and 1–26 introns in AGO genes of chickpea, pigeonpea, and groundnut, respectively. Members of AGO I and AGO IV clade were identified with higher number of introns as compared to AGO II members in all three legumes. These gene structures were similar to the ones found in rice (Kapoor et al., 2008). AGOs are endonucleatically active due to the presence of a conserved metal-chelating Asp-Asp-His (DDH) motif in their PIWI domain. Along with DDH (D760, D845, H986), one more conserved residue H798 of AtAGO1 is also known to play an important role in AGO slicer activity. However, studies on Arabidopsis, rice, maize and soybean reveals that DDH/H is not a quintessential motif. We also observed that in AGO proteins the DDH/H motif was substituted by patterns like DDD/H, DDH/P, DDH/S. Multiple sequence alignment of chickpea, pigeonpea, and groundnut AGOs with Arabidopsis AGOs unveiled the presence of conserved DDH/H motif in seven chickpea, eight pigeonpea, and nine groundnut (five *A. duranensis*, four *A. ipaensis*) AGOs (Table 4). It was seen that generally the members of the same clade exhibited the same patterns (Supplementary Figures 2A–C).

**Table 4:** **Patterns of DDH motif corresponding to D760, D845, and H986 of Arabidopsis AGO1 in chickpea, pigeonpea, and groundnut (*A. duranensis* and *A. ipaensis*)**.

**Chickpea**	**Motif**	**Pigeonpea**	**Motif**	***A. duranensis***	**Motif**	***A. ipaensis***	**Motif**
CaAGO1	DDH/H	CcAGO1	DDH/H	AdAGO1	DDH/H	AiAGO1	DDH/H
CaAGO3a	DDD/H	CcAGO3a	DDD/H	AdAGO3a	DDD/H	AiAGO3a	DDD/H
CaAGO3b	DDD/N	CcAGO3b	DDD/N	AdAGO3b	DDD/N	AiAGO3b	DDD/N
CaAGO4a	DDH/A	CcAGO4a	DDH/P	AdAGO4a	DDH/P	AiAGO4a	DDH/P
CaAGO4c	DDH/A	CcAGO4c	DDH/A	AdAGO4c	DDH/A	AiAGO4c	DDH/A
CaAGO5	DDH/H	CcAGO5	DDH/H	AdAGO5	DDH/H	AiAGO5	DDH/H
CaAGO6a	DDH/P	CcAGO6	DDH/S	AdAGO6	DDH/S	AiAGO6	DDH/S
CaAGO6b	DDH/P	–	–	–	–	–	–
CaAGO7	DDH/H	CcAGO7	DDH/H	AdAGO7	DDH/H	AiAGO7	DDH/H
–	–	CcAGO9	DDH/S	AdAGO9	DDH/P	AiAGO9	DDH/P
CaAGO10a	DDH/H	CcAGO10a	DDH/H	AdAGO10a	DDH/H	AiAGO10a	DDH/H
CaAGO10c	DDH/Q	CcAGO10c	DDH/H	–	–	–	–
CaAGO10d	DDH/H	CcAGO10d	DDH/H	–	–	–	–
CaAGO10e	DDH/H	–	–	–	–	–	–
–	–	CcAGO10f	DDH/H	AdAGO10f	DDH/H	AiAGO10f	DD-/H

All RDR genes are known to have a conserved RdRP domain. However, clade I and clade II in the legumes studied here have an additional conserved RNA Recognition Motif (RRM) as also seen in clades 1 and 2 of Arabidopsis and soybean. In RDRs, among all the motifs analyzed, motif 2 in chickpea and pigeonpea, and motif 6 in groundnut was seen to be conserved among all the RDR members. A short sub-sequence of this motif, DLDGD was seen to be conserved in all RDR clades except clade III where it got converted to DFDGD. This motif is seen conserved in other species as well, and corresponds to the catalytic β′ subunit of DNA-dependent RNA polymerases (Zong et al., 2009). Another motif, PCLHPGD[V/I]R was seen to be conserved in all RDR clades except RDR III, where it got converted to PGLHFGDIH in all three legumes. The gene structure analysis identified introns ranging from 1 to 16 in chickpea, 2 to 17 in pigeonpea, 3to 17 in *A. duranensis*, and 2 to 18 in *A. ipaensis*. Interestingly, clade III RDRs were seen to have the maximum number of introns in all three legumes (Table 3, Supplementary Figures 3A–C). The motif analysis suggests a strong conservation of DCL, AGO, and RDR sequences among the studied legumes. However, the differences among the clades of a same protein family indicate the non-redundant structure and function of members of different clades of a family.

### Chromosomal localization and gene duplication

**Figure 2:**
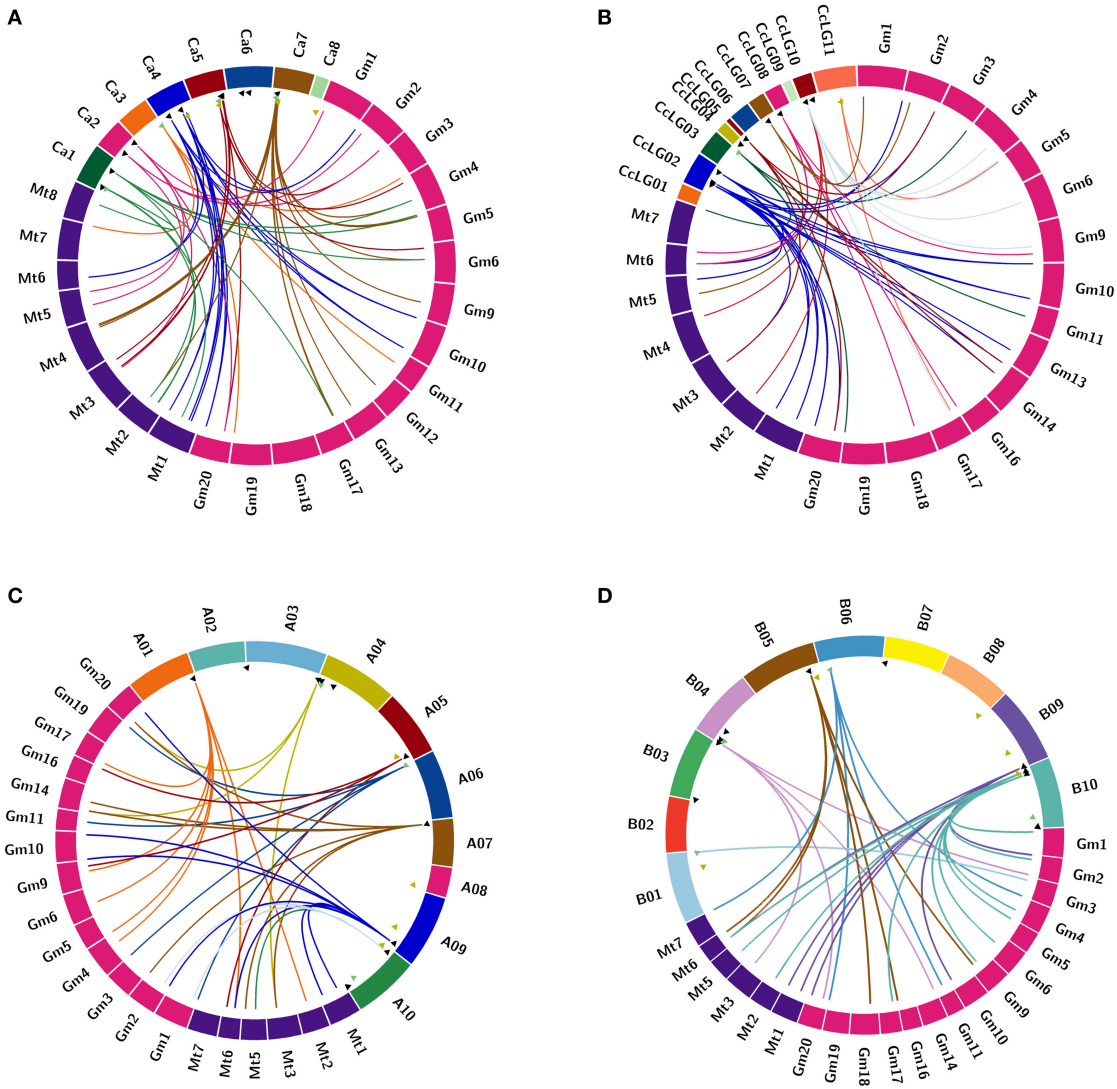
**Comparative analysis of orthologus relationship of sRNA biogenesis genes in (A)** chickpea; **(B)** pigeonpea; **(C)** *A. duranensis*; and **(D)** *A. ipaensis* with Medicago and soybean. Orthology among genes is depicted using circos. The triangles represent the chromosomal location of the respective genes. Triangles in green, black and yellow denote DCL, AGO, and RDR, respectively. Strokes originating from these triangles represent the orthologous genes present in Medicago and soybean.

Chickpea DCLs were distributed on pseudomolecules Ca1, Ca3, Ca5, and Ca7 whereas in pigeonpea, only CcDCL1 was found to be present on pseudomolecule CcLG03 while rest of the DCLs were scattered on unassembled scaffolds (Table 1). All AGO genes except CaAGO1 in chickpea were found to be uniformly distributed on all eight pseudomolecules. Similarly, in the case of pigeonpea, most of the AGO genes (10) were identified on pseudomolecules with a maximum number of genes on pseudomolecule CcLG02 (Table 2). In chickpea, two RDRs—CaRDR1a and CaRDR1b were located on pseudomolecule Ca4. The other RDRs, CaRDR2, CaRDR3, and CaRDR6, were positioned on pseudomolecule Ca5, Ca7, and Ca8, respectively. In pigeonpea, only CcRDR2 was placed on pseudomolecule CcLG11; all other RDRs were scattered on scaffolds. In groundnut, all the DCLs, AGOs, and RDRs were distributed on pseudomolecules (Tables 1–3, Figures 2A–D).

Gene duplication underlines the phenomenon of evolution as it functions as a template for subsequent modifications acted upon by natural selection. Gene families are nothing but true paralogs resulting from gene duplication events. In this study, we identified the duplicated events of sRNA biogenesis genes in the genomes of three SAT legumes. A paralog for an identified gene was considered only if it retained at least one of all the essential domains responsible for defining that gene to be a part of a specific family. Considering the above criteria, no duplicated DCL gene was found in chickpea, whereas two were found in pigeonpea. Likewise, one duplicated DCL gene was found each in *A. duranensis* and *A. ipaensis*. In chickpea and pigeonpea, three and four pairs of duplicated AGO genes were found, respectively. In groundnut, *A. duranensis* harbored one, whereas *A. ipaensis* shared two events of duplication of the same AGO gene. No duplication was observed for RDR genes in both chickpea and pigeonpea. By contrast, in groundnut, two pairs of duplicated RDR genes were identified for each of the two groundnut sub genomes (Supplementary Table 2).

The orthologs of DCL, AGO, and RDR proteins of chickpea, pigeonpea, and groundnut were identified in two closely related legumes, Medicago, and soybean (Figures 2A–D). Chickpea was seen to share the maximum orthologs with Medicago and pigeonpea with soybean. In the case of groundnut, DCL and AGO orthologs were present in Medicago and soybean, whereas no RDR orthologs were seen in any of the two legumes (Supplementary Table 3). These findings can be correlated with the divergence of these legume species during the course of evolution. For instance, chickpea and Medicago belong to the same clade, i.e., Galegoid, pigeonpea and soybean belong to the Phaseoloid clade while groundnut belongs to the Dalbergioid clade.

### Promoter analysis, miRNA identification, annotation, and interaction networks

Promoter analysis identified a number of *cis*-acting elements in all members of DCL, AGO, and RDR of all three legumes. Light responsive elements were the most frequent followed by those related to stress responses and hormones (Supplementary Tables 4–6). Prevalence of a high number of light responsive elements prompts the role of these genes in regulation of photoperiodic control of flowering; however, this would require further validation for arriving at a conclusion. Regulatory elements corresponding to endosperm expression, and circadian control were also seen. *Cis*-acting elements relating to drought, heat, and low-temperature stress suggest the possible role of these genes in regulating the expression in response to these stresses. The presence of hormones, such as ABA, ethylene, and gibberellin related elements prompt the involvement of these genes in signaling pathways as well.

All the DCL genes in chickpea, pigeonpea, and groundnut were recognized as miRNA targets. The DCL1 gene irrespective of the legume species studied in this study was found to be targeted by miR162. One of the miRNAs, miR1515 involved in hyper nodulation in soybean is known to target DCL2 (Li et al., 2010). The same miRNA was found to be targeting CcDCL2, AdDCL2, and AiDCL2 in this study (Supplementary Table 7). A total of 10, 11, 8, and 8 AGO genes were identified as targets for miRNAs in chickpea, pigeonpea, *A. duranensis*, and *A. ipaensis*, respectively. It has been reported that in Arabidopsis, the co-regulation of AGO1 and miR168 is indispensable for normal miRNA functioning and plant development (Vaucheret et al., 2004). AGO1 of chickpea and groundnut (both *A. duranensis* and *A. ipaensis*) was also seen to be targeted by miR168 substantiating the indispensability of miR168 in plant growth and regulation. However, in pigeonpea this pattern was not observed (Supplementary Table 8). In case of RDRs, a total of 2, 4, 4, and 3 RDR genes were identified as the targets for miRNAs in chickpea, pigeonpea, *A. duranensis*, and *A. ipaensis*, respectively. miR156, one of the stress responsive miRNAs (Stief et al., 2014), was seen to target a wide range of genes which include AGO genes in groundnut, and RDR genes in chickpea and pigeonpea suggesting the involvement of various mechanisms in sRNA regulation (Supplementary Table 9).

DCL, AGO, and RDR are broadly known to facilitate the sRNA biogenesis. However, each of them has a precise role in the molecular and biological modus operandi of sRNA biogenesis and regulation of gene expression. Annotation of the identified respective family members revealed that clade IV DCLs were involved in a biological process of mitotic cell cycle and virus induced gene silencing (VIGS; Supplementary Table 10). Among AGOs, clade IV AGO6 was found to be involved in siRNA binding. Clade II and VI RDRs together were involved in VIGS; however, clade II RDR was identified to play a role in DNA methylation and response to fungus (Supplementary Tables 11, 12). Only clade II RDRs showed antifungal response in all three legumes (Supplementary Table 12). *In-silico* protein interactions among the members of DCL, AGO, and RDR showed very strong associations among themselves suggesting that all three proteins interact with each other to accomplish the task of RNA interference (Supplementary Figures 4A,C,E). Along with this, these proteins also showed strong interactions among themselves in response to stress (Supplementary Figures 4B,D,F). It was seen that most of the interacting partners, such as HYL1, NRPD, SE of these families were common in all three species. These interactions indicate the role of these proteins in processing the pri-miRNA into miRNA in the light of the fact that DCL1, SE, TOUGH, and HYL1 in Arabidopsis are well-known to form a complex to process pri-miRNA into miRNA duplex in the nucleus (Fang and Spector, 2007; Song et al., 2007).

### Gene expression analysis in response to biotic stresses

#### Ascochyta blight (AB) infection in chickpea

**Figure 3:**
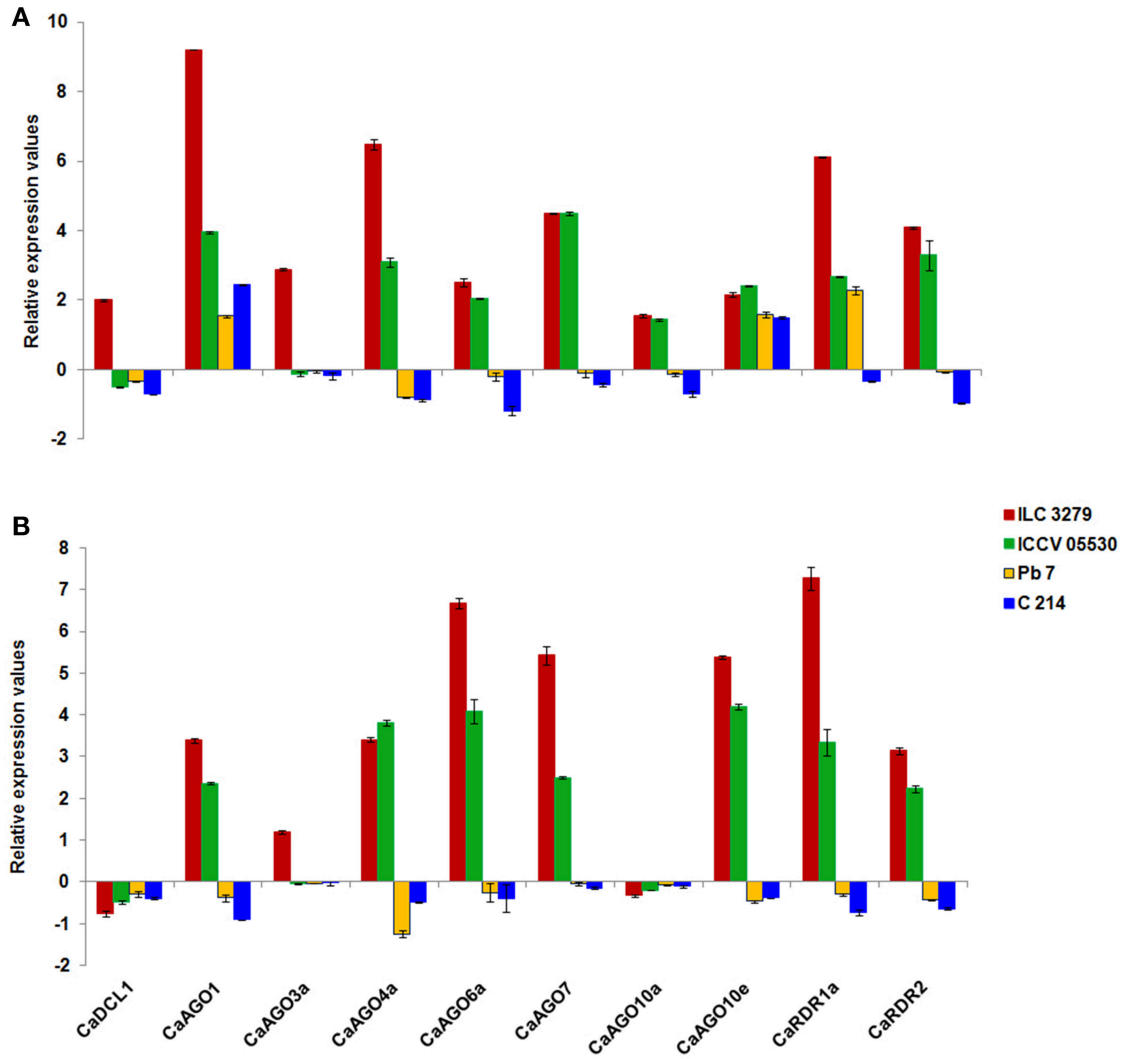
**Expression profiling of DCL, AGO, and RDR genes in leaf tissues of chickpea in response to AB with respect to its control at (A)** 7th dpi; **(B)** 11th dpi.

In order to understand the role of DCL, AGO, and RDR in post-transcriptional regulation of gene expression in response to AB, we randomly selected 10 candidate genes belonging to DCL (1), AGO (7), and RDR (2) family. At 7th dpi, both moderately resistant genotypes (ILC 3279 and ICCV 05530) showed a general trend of upregulation for all the ten genes, except CaDCL1 and CaAGO3a, which were found to be downregulated in ICCV 05530. The susceptible genotypes (Pb 7 and C 214) showed a general trend of downregulation, except CaAGO1 and CaAGO10e (Figure 3A). We observed an upregulation of clade I AGO genes (CaAGO1 and CaAGO10e) in all four genotypes irrespective of resistance/susceptibility at 7th dpi. However, on 11th dpi, these genes showed upregulation in resistant and downregulation in susceptible genotypes (Figure 3B) implying their role in resistance. RDR2 was shown to positively regulate defense responses against fungal pathogen, *Verticillium dahliae* in Arabidopsis (Ellendorff et al., 2009). Also, the *rdr2* mutants displayed enhanced susceptibility to Verticillium wilt disease compared with wild-type plants. In this study, we also observed increased CaRDR2 expression in resistant genotypes and decreased expression in susceptible genotypes at both stages of infection implying the role of RDR2 in imparting resistance/sensitivity to AB responsive chickpea genotypes. Furthermore, a similar trend of increased and decreased expression of CaAGO7 gene in resistant and susceptible genotypes, respectively, was observed. Enhanced susceptibility in Arabidopsis to the fungus (Ellendorff et al., 2009) in *ago7* mutant suggests a positive role of AGO7 in anti-fungal defense. AGO4 mutations are known to render plants more susceptible to necrotrophic fungal pathogens (Seo et al., 2013). Therefore, increased expression of CaAGO4 can be associated with resistance against the necrotrophic fungi, *Ascochyta rabiei* in chickpea. The expression profile of DCL, AGO, RDR genes observed in this study along with previous findings throw light on the involvement of these genes and their products (sRNAs) in combating AB stress in chickpea.

#### Sterility mosaic disease (SMD) infection in pigeonpea

**Figure 4:**
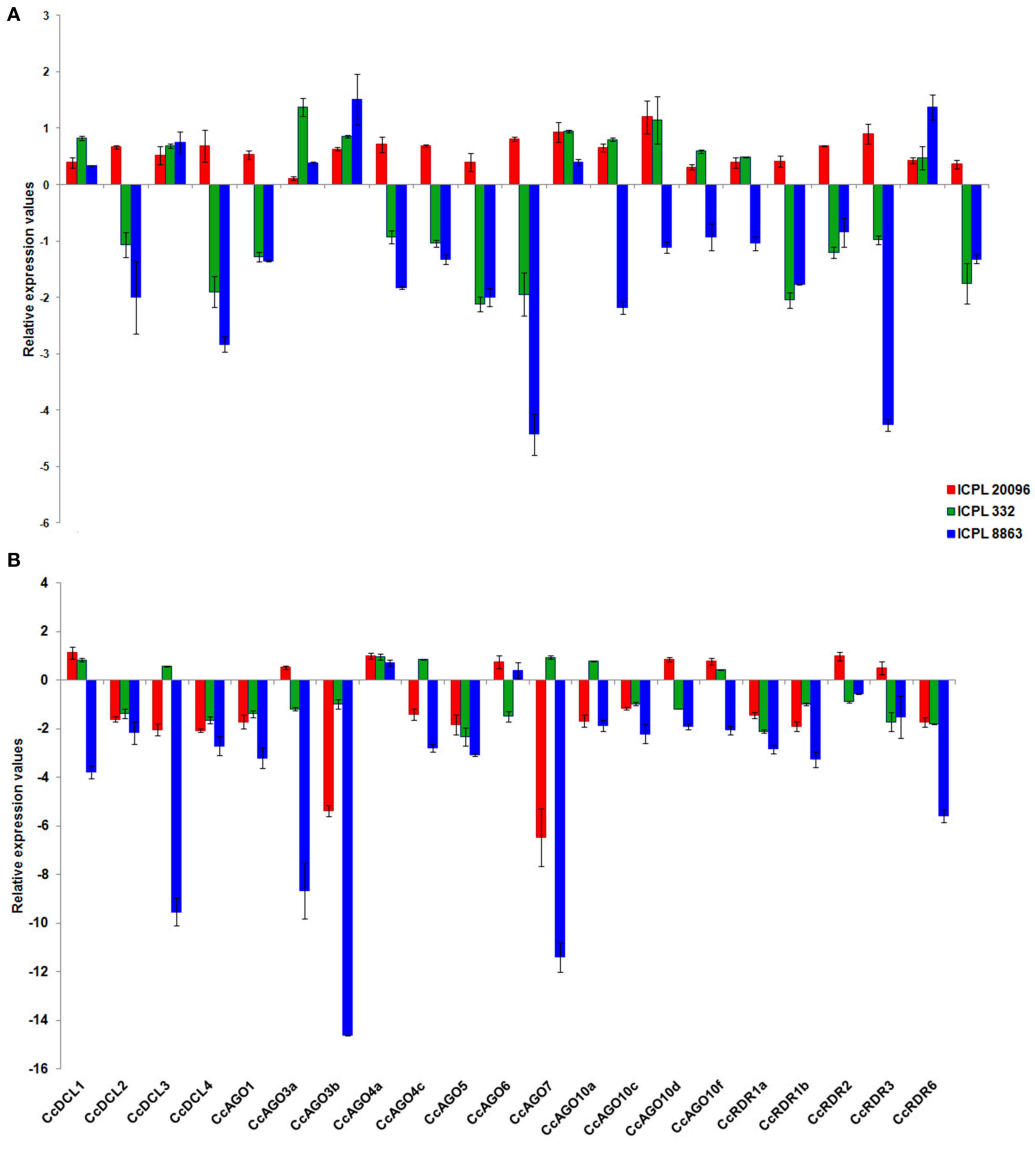
**Expression profiling of DCL, AGO, and RDR genes in leaf tissues of pigeonpea in response to SMD with respect to its control at (A)** 7th dpi; **(B)** 14th dpi.

In order to investigate the role of sRNA biogenesis genes in viral defense, we studied the expression profile of 21 genes including DCLs (4), AGOs (12), and RDRs (5). The expression of these genes in response to SMD was studied using qRT-PCR at initial (7th dpi) and severe stress (14th dpi) in a resistant (ICPL 20096), a susceptible (ICPL 332), and a highly susceptible (ICPL 8863) genotype of pigeonpea. Significantly, all DCL, AGO, and RDR genes were found to be upregulated at 7th dpi in resistant genotype. General pattern of downregulation of these genes in highly susceptible pigeonpea genotype was observed. A total of 11 out of 21 genes showed stark downregulation of expression in both the susceptible genotypes compared to the resistant ones at 7th dpi, as expected. However, clade III genes of DCL, RDR and clade II of AGO at 7th dpi were found to be induced in all genotypes, along with clade I DCL. At 14th dpi, most of the genes (13) in resistant genotype showed downregulation that were upregulated on 7th dpi. As the infection progressed (14th dpi), all genes, except CcAGO4a and CcAGO6 were seen to be downregulated in a highly susceptible genotype. Interestingly, four genes CcAGO7, CcDCL3, CcAGO3b, and CcAGO3a showed a profound dip in expression, which antagonistically showed an upregulated expression at initial stage of infection in the highly susceptible genotype (Figure 4A). During the severe expression state, an overall trend of downregulation of sRNA biogenesis genes was observed, except a few genes that were slightly upregulated.

We also observed an up and downregulated expression of CcDCL2 and CcDCL4 at 7th dpi in resistant and susceptible genotypes. However, at 14th dpi (severe stress) both genes showed downregulation irrespective of the genotypes, which could be attributed to senescence and programed cell death (PCD) during the penultimate days of a plant's life cycle. Considering the findings that loss-of-function mutation in DCL2 and DCL4 is necessary and sufficient to make plants susceptible to single stranded viruses (Deleris et al., 2006), it can be inferred that an increased DCL2 and DCL4 expression in resistant, and decreased expression in susceptible pigeonpea genotypes at 7th dpi may play an important role in resistance and susceptibility, respectively (Figure 4B). As observed in the case of CcDCL2 and CcDCL4, a similar trend of upregulated expression of CcRDR1a and CcRDR1b in resistant genotype and downregulated expression in susceptible genotype at 7th dpi was observed. RDR1 has been demonstrated to fight against viral infection (TMV and potato virus X) through post-transcriptional gene silencing pathway (Yu et al., 2003). In accordance with the findings of RDR involvement in viral defense, we also observed an upregulated expression of CcRDRs in resistant genotypes at 7th dpi against SMD viral invasion, thus substantiating the role of RDRs against viral pathogen in pigeonpea at an early stage of infection. The collinearity in the expression of AGOs, RDRs, and DCLs in resistant genotypes at 7th dpi can also be correlated with the strong association identified among these proteins in response to stress (Figure 4A and Supplementary Figure 4D).

#### Rust and LLS infection in groundnut

**Figure 5:**
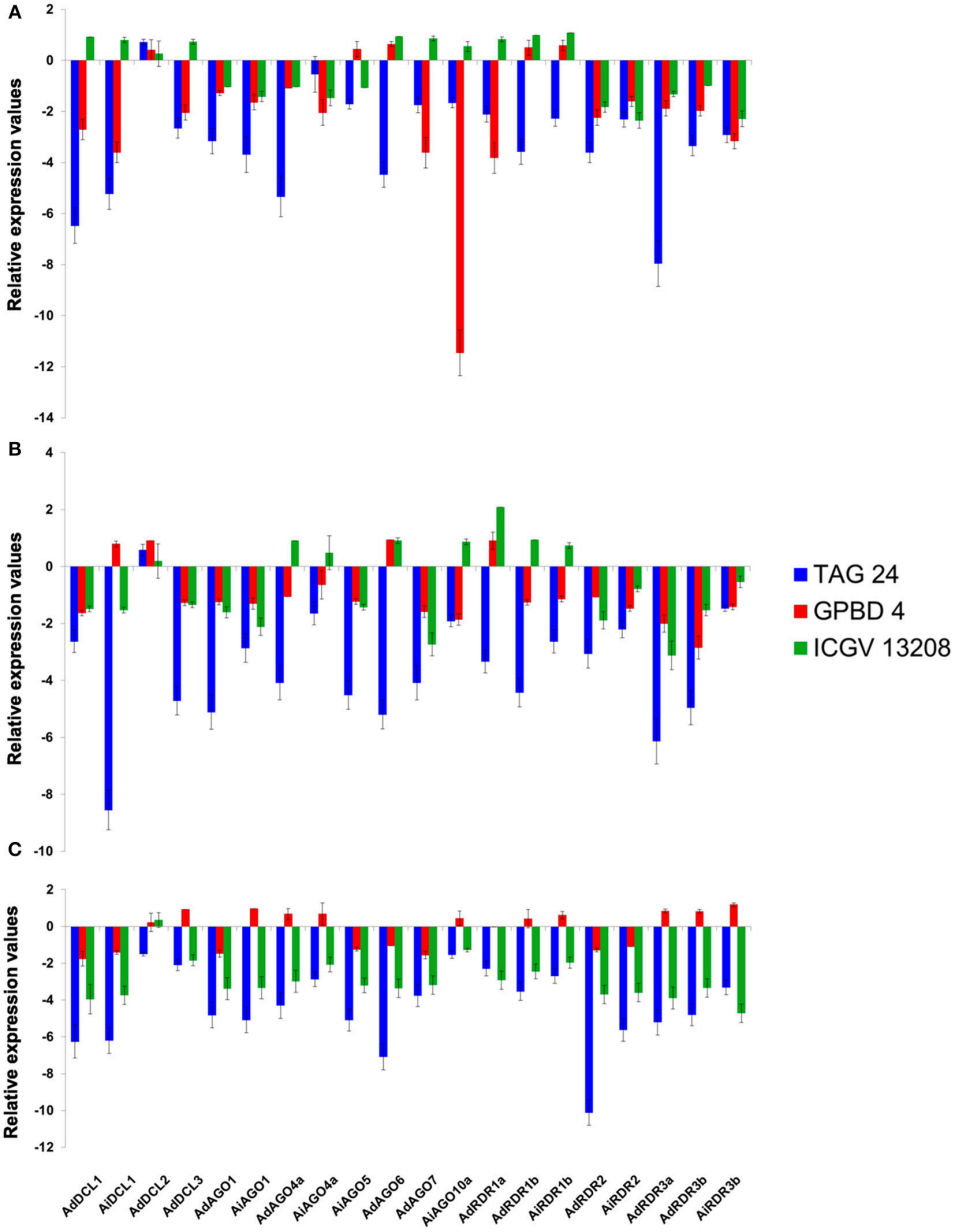
**Expression profiling of DCL, AGO, and RDR genes in leaf tissues of groundnut in response to Rust and LLS with respect to its control at (A)** 21st dpi; **(B)** 35th dpi; and **(C)** 50th dpi.

Expression analysis of randomly selected DCL (4), AGO (8), and RDR (8) genes in rust and LLS responsive genotypes of groundnut revealed their role in response to stress imposed by their causal pathogens. Expression studies were conducted at 21st dpi (inception of infection), 35th dpi (systemic spread of infection), and 50th dpi (terminal stage of infection). In course of time, all the genes, except AdDCL2, were consistently downregulated in TAG 24 (susceptible). However, GPBD 4 (resistant) showed an inconsistent expression pattern with a stint of induced expression at all time points. A few genes (4–5) showed upregulation at 21st and 35th dpi, whereas at 50th dpi nearly half of the genes (11) got upregulated in GPBD 4. Out of all the upregulated genes at 50th dpi, AiAGO10a, AdAGO4a, AiAGO4a, clade III RDRs, AdDCL3 showed an initial downregulation at 21st and 35th dpi. AiAGO10a, in particular, showed highly repressed levels at 21st dpi as compared to other genes. Interestingly, in the case of ICGV 13208 a pattern of gradual downregulation of gene expression was observed. At 21st dpi half (10) of the genes were downregulated followed by repression of 12 genes at 35th dpi, and at 50th dpi all the genes except AdDCL2 were downregulated. AdDCL2 showed an upregulation at 21st and 35th dpi in all three genotypes. However, at 50th dpi it showed downregulation in susceptible genotype, which could be attributed to senescence as a result of PCD during the later stages of the plant's life cycle (Figures 5A–C). Also, it is to be noticed that the 50th dpi (85th day of groundnut's life cycle) is well-past its reproductive stage at which its ability to combat stress responses is highly compromised.

There have been very limited studies of expression of sRNA biogenesis genes in response to fungal pathogen and those studies have largely demonstrated that the expression of these genes is directly proportional to the resistance of the genotype in response to pathogen attack. In the current study, chickpea and pigeonpea showed similar expression pattern of upregulation in resistant genotype, contradicted by groundnut where resistant genotypes showed downregulation for most of the genes. Susceptible genotypes of all three legumes showed a general pattern of downregulation across different stages of both viral and fungal stress. The contrasting expression patterns in groundnut can be due to the fact that the genome-wide survey was carried out in diploid progenitors of groundnut, and expression analysis was performed on cultivated tetraploids. However, it definitely requires further substantial evidence to elucidate the role of epigenetic pathways operating under such stress in tolerant and susceptible genotypes. It can be inferred from this observation that the sRNA biogenesis machinery gets affected, leading to its highly depleted levels during the terminal stages of the plant's life.

## Conclusion

This is the first report on genome-wide identification and characterization of sRNA biogenesis proteins in chickpea, pigeonpea, and groundnut. The number of proteins in each family (DCL, AGO, and RDR) were found to be conserved across all three legumes. Phylogenetic analysis revealed clade and species-specific differences within members of sRNA biogenesis proteins which might reflect their functional divergence. Further, comparative analysis of DCLs, AGOs, and RDRs reflected the prevalence of functional overlapping and compensatory phenomenon in legumes to accomplish the role of these genes by other homologs. Upstream regulatory regions of the sRNA biogenesis genes were marked by the presence of stress hormone related elements. Moreover, differential expression of these genes under biotic stress confirmed their involvement in combating stress. Expression studies under biotic stress also revealed species-specific expression of these genes. Overall, our findings are an indication of structural diversification of DCL, AGO, and RDR and divergences of the studied legumes during the course of evolution. The genes reported in this study can be targeted in the near future for gene manipulation through post-transcriptional gene silencing approaches so as to develop resistant cultivars against biotic stresses in these legumes.

## Author contributions

RKV conceived and designed the experiment. VG, GA, LTP, SNN, HK, MS, and PBK performed the experiments. VG, AWK, and DD analyzed the data. VG and GA with support of RKV wrote the manuscript.

### Conflict of interest statement

The authors declare that the research was conducted in the absence of any commercial or financial relationships that could be construed as a potential conflict of interest.

## Supplementary material

Supplementary Figure 1 **Identification and characterization of domains and motifs, exon-intron structure of the identified DCL genes in (A)** chickpea; **(B)** pigeonpea; and **(C)** groundnut (*A. duranensis* and *A. ipaensis*). In domain organization, roman numbers I, II, III, IV, V, and VI represent DEAD, Helicase-C, Dicer-dimer, PAZ, RNAase III, and dsrm domain, respectively.

Supplementary Figure 2 **Identification and characterization of domains and motifs, exon-intron structure of the identified AGO genes in (A)** chickpea; **(B)** pigeonpea; and **(C)** groundnut (*A. duranensis* and *A. ipaensis*).

Supplementary Figure 3 **Identification and characterization of domains and motifs, exon-intron structure of the identified RDR genes in (A)** chickpea; **(B)** pigeonpea; and **(C)** groundnut (*A. duranensis* and *A. ipaensis*).

Supplementary Figure 4 **Interaction network of DCL, AGO, and RDR proteins in (A,B)** chickpea; **(C,D)** pigeonpea; and **(E,F)** groundnut (*A. duranensis* and *A. ipaensis*). In **(A,C,E)**, thicker lines suggest stronger associations while in **(B,D,F)** red colored nodes indicate genes involved in defense response.

Supplementary Table 1 **List of primers used for qRT-PCR studies in chickpea, pigeonpea, and groundnut**.

Supplementary Table 2 **List of paralogs of the identified DCL, AGO, and RDR genes in chickpea, pigeonpea, and groundnut (*A. duranensis* and *A. ipaensis*)**.

Supplementary Table 3 **List of orthologs of the identified DCL, AGO, and RDR genes in chickpea, pigeonpea, and groundnut (*A. duranensis* and *A. ipaensis*) in Medicago and soybean**.

Supplementary Table 4 **List of the *cis*-regulatory elements identified upstream of the DCL genes in chickpea, pigeonpea, and groundnut (*A. duranensis* and *A. ipaensis*)**.

Supplementary Table 5 **List of the *cis*-regulatory elements identified upstream of the AGO genes in chickpea, pigeonpea, and groundnut (*A. duranensis* and *A. ipaensis*)**.

Supplementary Table 6 **List of the *cis*-regulatory elements identified upstream of the RDR genes in chickpea, pigeonpea, and groundnut (*A. duranensis* and *A. ipaensis*)**.

Supplementary Table 7 **Identification of miRNAs targeting the DCL genes in chickpea, pigeonpea, and groundnut (*A. duranensis* and *A. ipaensis*)**.

Supplementary Table 8 **Identification of miRNAs targeting the AGO genes in chickpea, pigeonpea, and groundnut (*A. duranensis* and *A. ipaensis*)**.

Supplementary Table 9 **Identification of miRNAs targeting the RDR genes in chickpea, pigeonpea, and groundnut (*A. duranensis* and *A. ipaensis*)**.

Supplementary Table 10 **Gene ontology annotation of the DCL genes in chickpea, pigeonpea, and groundnut (*A. duranensis* and *A. ipaensis*)**.

Supplementary Table 11 **Gene ontology annotation of the AGO genes in chickpea, pigeonpea, and groundnut (*A. duranensis* and *A. ipaensis*)**.

Supplementary Table 12 **Gene ontology annotation of the RDR genes in chickpea, pigeonpea, and groundnut (*A. duranensis* and *A. ipaensis*)**.
