# Supplementary figures and images for: Genome-Wide Identification, Characterization, and Expression Analysis of Small RNA Biogenesis Purveyors Reveal Their Role in Regulation of Biotic Stress Responses in Three Legume Crops
Source: Front Plant Sci. 2017 Apr 25;8:488. doi: 10.3389/fpls.2017.00488 (PMC5404147; doi:10.3389/fpls.2017.00488)

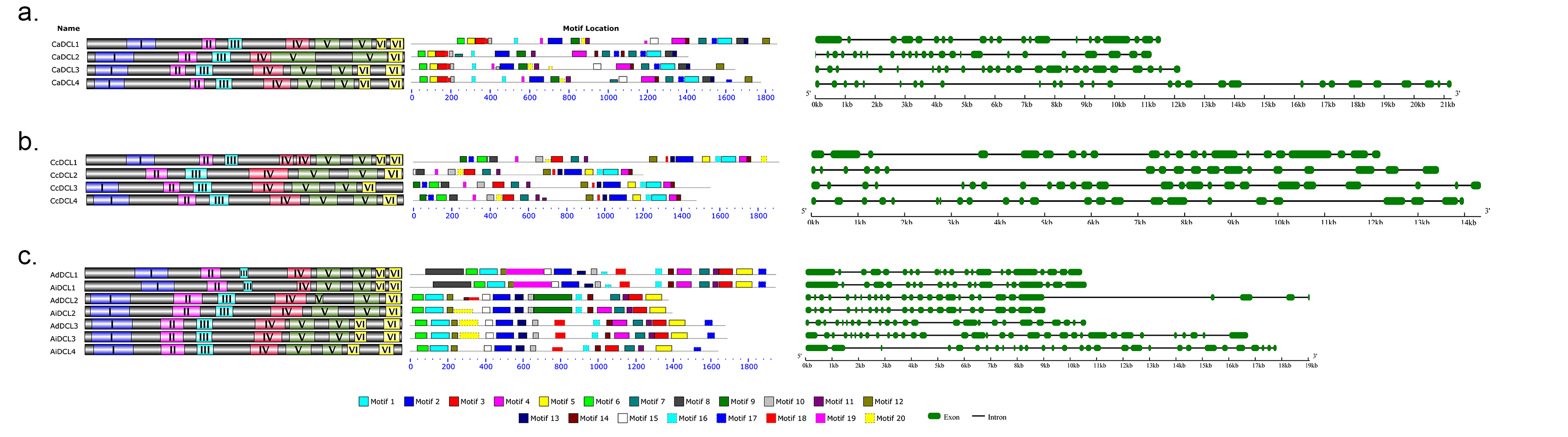

Supplement: Supplementary Figure 1 — Identification and characterization of domains and motifs, exon-intron structure of the identified DCL genes in (A) chickpea; (B) pigeonpea; and (C) groundnut (A. duranensis and A. ipaensis). In domain organization, roman numbers I, II, III, IV, V, and VI represent DEAD, Helicase-C, Dicer-dimer, PAZ, RNAase III, and dsrm domain, respectively. [file Image1.TIF]

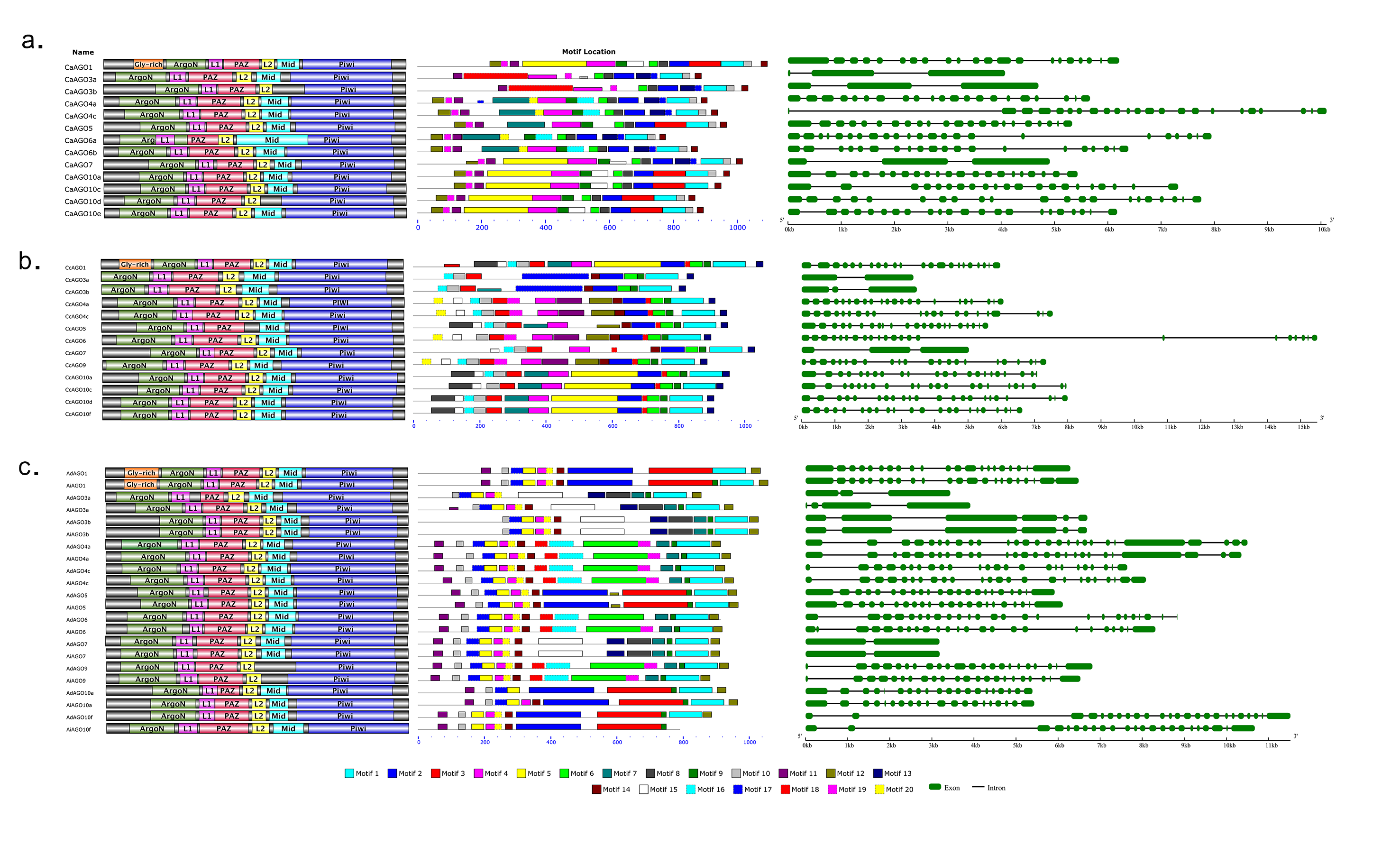

Supplement: Supplementary Figure 2 — Identification and characterization of domains and motifs, exon-intron structure of the identified AGO genes in (A) chickpea; (B) pigeonpea; and (C) groundnut (A. duranensis and A. ipaensis). [file Image2.TIF]

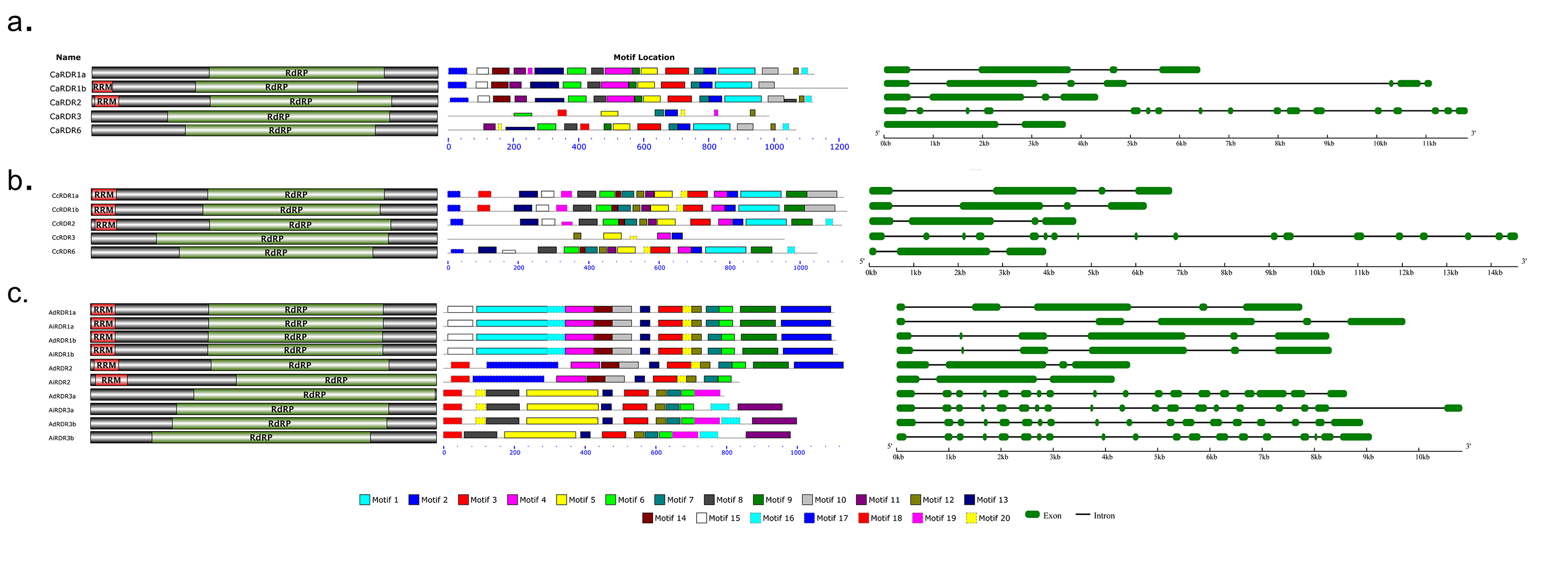

Supplement: Supplementary Figure 3 — Identification and characterization of domains and motifs, exon-intron structure of the identified RDR genes in (A) chickpea; (B) pigeonpea; and (C) groundnut (A. duranensis and A. ipaensis). [file Image3.TIF]

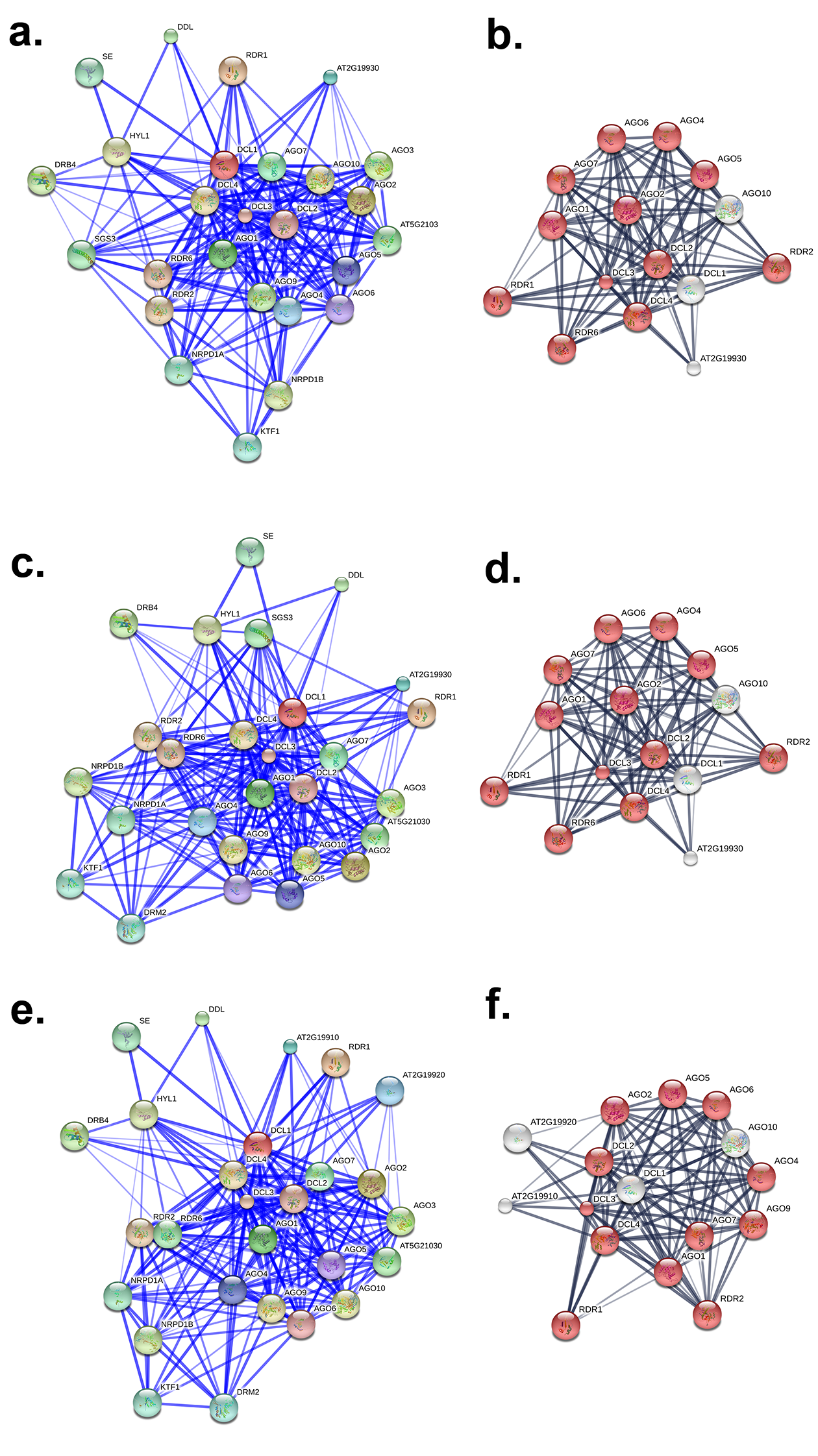

Supplement: Supplementary Figure 4 — Interaction network of DCL, AGO, and RDR proteins in (A,B) chickpea; (C,D) pigeonpea; and (E,F) groundnut (A. duranensis and A. ipaensis). In (A,C,E), thicker lines suggest stronger associations while in (B,D,F) red colored nodes indicate genes involved in defense response. [file Image4.TIF]
